# Exploring the Potential of a Normalized Hotspot Index in Supporting the Monitoring of Active Volcanoes Through Sea and Land Surface Temperature Radiometer Shortwave Infrared (SLSTR SWIR) Data

**DOI:** 10.3390/s25061658

**Published:** 2025-03-07

**Authors:** Alfredo Falconieri, Francesco Marchese, Emanuele Ciancia, Nicola Genzano, Giuseppe Mazzeo, Carla Pietrapertosa, Nicola Pergola, Simon Plank, Carolina Filizzola

**Affiliations:** 1 Institute of Methodologies for Environmental Analysis, National Research Council, 85050 Tito Scalo, Italy; alfredo.falconieri@cnr.it (A.F.); emanuele.ciancia@cnr.it (E.C.); giuseppe-mazzeo@cnr.it (G.M.); carla.pietrapertosa@cnr.it (C.P.); nicola.pergola@cnr.it (N.P.); carolina.filizzola@cnr.it (C.F.); 2 Department of Architecture, Built Environment and Construction Engineering (ABC), Politecnico di Milano Via Ponzio 31, 20133 Milan, Italy; nicola.genzano@polimi.it; 3 German Aerospace Center DLR, German Remote Sensing Data Center, 82234 Oberpfaffenhofen, Germany; simon.plank@dlr.de

**Keywords:** volcanoes, normalized hotspot indices, SLSTR, thermal anomalies

## Abstract

Every year about fifty volcanoes erupt on average, posing a serious threat for populations living in the neighboring areas. To mitigate the volcanic risk, many satellite monitoring systems have been developed. Information from the medium infrared (MIR) and thermal infrared (TIR) bands of sensors such as the Moderate Resolution Imaging Spectroradiometer (MODIS) and the Visible Infrared Imaging Radiometer Suite (VIIRS) is commonly exploited for this purpose. However, the potential of daytime shortwave infrared (SWIR) observations from the Sea and Land Surface Temperature Radiometer (SLSTR) aboard Sentinel-3 satellites in supporting the near-real-time monitoring of thermal volcanic activity has not been fully evaluated so far. In this work, we assess this potential by exploring the contribution of a normalized hotspot index (NHI) in the monitoring of the recent Home Reef (Tonga Islands) eruption. By analyzing the time series of the maximum NHI_SWIR_ value, computed over the Home Reef area, we inferred information about the waxing/waning phases of lava effusion during four distinct subaerial eruptions. The results indicate that the first eruption phase (September–October 2022) was more intense than the second one (September–November 2023) and comparable with the fourth eruptive phase (June–August 2024) in terms of intensity level; the third eruption phase (January 2024) was more difficult to investigate because of cloudy conditions. Moreover, by adapting the NHI algorithm to daytime SLSTR SWIR data, we found that the detected thermal anomalies complemented those in night-time conditions identified and quantified by the operational Level 2 SLSTR fire radiative power (FRP) product. This study demonstrates that NHI-based algorithms may contribute to investigating active volcanoes located even in remote areas through SWIR data at 500 m spatial resolution, encouraging the development of an automated processing chain for the near-real-time monitoring of thermal volcanic activity by means of night-time/daytime Sentinel-3 SLSTR data.

## 1. Introduction

In the last 10,000 years more than 1500 volcanoes, mostly distributed along tectonic plate boundaries, have erupted [1]. Volcanic eruptions can cause fatalities even at a great distance from the source (e.g., because of lahars and induced tsunamis), destroy infrastructures, and affect the environment and climate when large amounts of volcanic ash and gases (e.g., sulfur dioxide, carbon dioxide) are injected into the atmosphere [2].

The effective monitoring of active volcanoes is, therefore, crucial to mitigate their impact on populations and surrounding areas (e.g., [1,2,3]).

Satellite observations over decades provide an important contribution in this direction, enabling the monitoring of volcanoes even in remote and inaccessible areas in which traditional surveillance systems are often missing (e.g., [4]).

The MODIS (Moderate Resolution Imaging Spectroradiometer) sensor, aboard the Terra and Aqua satellites, has extensively been used to analyze the thermal manifestations of active volcanoes, thanks to channels in the MIR (mid-infrared) and TIR (thermal infrared) bands sensitive to the active magmatic features, a spatial resolution of 1 km at the nadir, and the daily temporal coverage (e.g., [4,5,6,7]). The availability of an MIR channel with a high saturation temperature (around 500 K) was then exploited to better estimate the FRP (fire radiative power), i.e., the rate of energy emitted by the fire [8]. This parameter is usually indicated as the VRP (volcanic radiative power) in reference to volcanic activity (e.g., [9,10]).

Recently, Coppola and co-authors presented an extended study investigating the annual trends of VRE (volcanic radiative energy), i.e., the energy radiated by volcanic thermal features, retrieved from infrared MODIS data [4]. Nonetheless, MODIS is close to its end of life. Indeed, Terra-MODIS will remain operational until the end of the mission in December 2025 [11]. Both VIIRS (Visible Infrared Imaging Radiometer Suite), onboard the Suomi NPP (NASA/NOAA Suomi National Polar-orbiting Partnership) and NOAA-20/21 satellites, and SLSTR (Sea and Land Surface Temperature Radiometer), which is one of the instruments of the Sentinel-3 platforms, guarantee the continuity of observations of the active volcanic areas through infrared data at a moderate spatial resolution (up to 375 m for VIIRS) (e.g., [11,12,13,14]).

FIRMS (Fire Information for Resource Management System [15]) is a satellite-based system providing global hotspot products from MODIS and VIIRS observations. These products were used also to investigate some recent and intense volcanic eruptions (e.g., [16,17,18]). Other systems such as the MODVOLC [5] and the MIROVA (Middle Infrared Observation of Volcanic Activity [10]), which was recently upgraded to perform with VIIRS data [11], were specifically developed to monitor active volcanoes. Multiplatform observing systems integrating SLSTR MIR observations were then developed (e.g., [19,20]).

Some earlier studies assessed the potential of SLSTR SWIR (shortwave infrared) observations in detecting high-temperature features (e.g., [21,22,23,24,25]). The Copernicus Sentinel-3 NRT (near-real-time) L2 (Level 2) FRP product, which is based on the algorithm proposed in [24], is available on a global scale and distributed by EUMETSAT [26,27]. Each detected fire is projected on the 1 km grid and matched with the corresponding MIR fire clusters when possible. The FRP-MIR value is then derived based on the matching distribution [27]. A comparison with the operational SLSTR FRP product was recently performed by other authors, analyzing the Stromboli and Etna (Italy) volcanoes [28]. On the other hand, further improvements in the identification of volcanic thermal features through daytime SLSTR SWIR data are possible.

Here, we assess this potential by using a normalized hotspot index to investigate the recent Home Reef (Tonga Islands) eruptions of 2022–2024. The index was originally proposed to map high-temperature features by means of Landsat 8/9 (L8/9) OLI/OLI2 (Operational Land Imager) and Sentinel-2 (S2) MSI (Multispectral Instrument) data [29].

Results from the daytime SLSTR SWIR observations are analyzed through comparison with the L2-FRP SLSTR and FIRMS-MODIS/VIIRS products and also assessed using information provided by ASTER (Advanced Spaceborne Thermal Emission and Reflection Radiometer), delivering infrared data at a higher spatial resolution (90 m in the TIR band).

An optimized NHI algorithm configuration tailored to SLSTR SWIR data is then, for the first time, proposed and tested, verifying its potential in integrating information about thermal anomalies over the Home Reef area from different satellite-based systems.

## 2. Test Case: Home Reef (Tonga Islands) Eruptions

**Figure 1:**
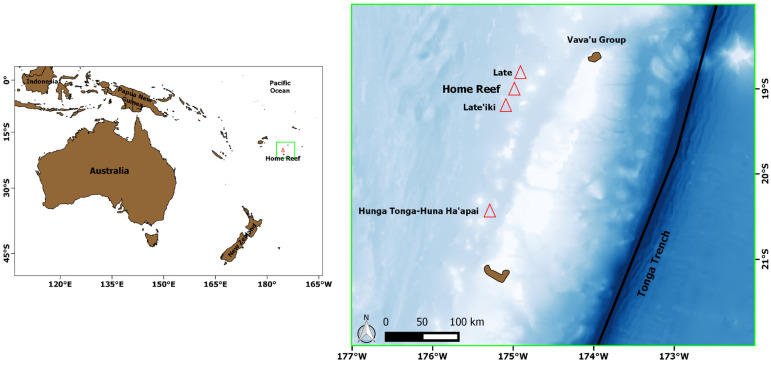
Geographic location of the Home Reef submarine volcano. In the background, the bathymetry from 9000 m (dark blue) to 200 m depth (light blue). Red triangles indicate main volcanoes; green square identifies the zoomed study area.

The Home Reef is a submarine volcano in the central islands of Tonga, located between Late’iki and Late Island (Figure 1). The first recorded Home Reef eruption took place in the mid-19th century. In 1984, another eruption occurred, leading to the formation of an ephemeral island and emitting an ash plume and a large volume of floating pumice [30]. During 2006, a submarine eruption rapidly evolved to subaerial activity upon the formation of another ephemeral island. In September 2022, after a period of intense water discoloration, a submarine eruption occurred. Lava effusion, ash plumes, discolored water, and gas-and-steam plumes were reported [31]. According to the Tonga Geological Service, during the eruption, the summit of the volcano rose above the sea level on 10 September 2022. The Sentinel-2 image of 9 September at 21:59 UTC (10 September 2022 at 12:59 LT) revealed the presence of a small island of about 70 m in diameter, together with the occurrence of a lava effusion, which was detected by satellite [30]. After the end of the first eruption phase, a new period of discolored plumes was recorded during November 2022–April 2023. On 12 May 2023, a sporadic eruption was observed by satellite, showing the presence of a less extended thermal anomaly. About four months later, the second eruption phase occurred, with a total of 11 eruptive events recorded during the period 12–17 October 2023 [31]. Afterward, the third and fourth eruption phases occurred in January 2024 and June–August 2024, respectively. Another eruption is in progress at the time of writing this paper [32], as indicated by the outputs of the NHI tool [33].

## 3. Data and Methods

### 3.1. SLSTR Instrument

**Figure 2:**
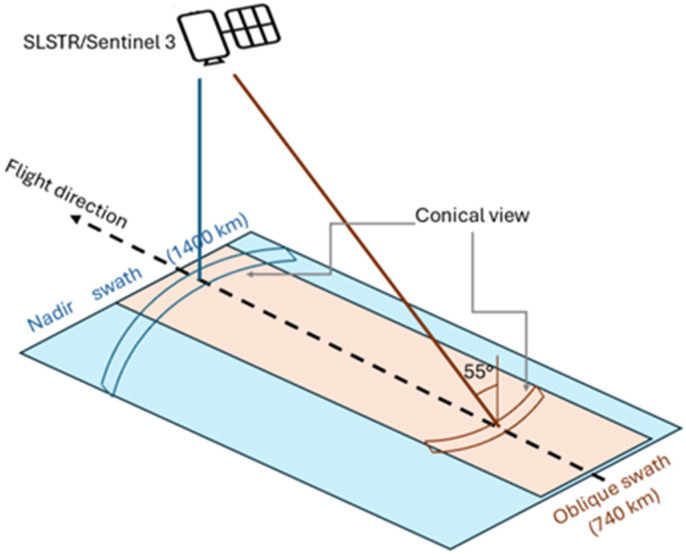
Scheme of SLSTR acquisition with nadir and oblique view (adapted from [35]).

The SLSTR sensor flies aboard the Sentinel-3A and Sentinel-3B satellites. The sensor is based on the conical imaging design of the earlier ATSR (Along Track Scanning Radiometer) instrument, with improved features. The instrument offers a dual view (oblique and nadir, with different swaths, see Figure 2), which is performed by two independent scan mirrors rotating in opposite directions [34]. The radiometer provides data from visible/near-infrared (VNIR) to thermal infrared in 9 spectral channels, with two additional bands (i.e., F-bands), which offer an increased dynamic range more suited for characterizing hot targets on the ground. The VIS-NIR and SWIR bands (i.e., S1–S6 bands) have a higher spatial resolution than the MIR/TIR ones (i.e., S7/S8-S9, F1/F2 bands) (see Table 1). A daily revisit time (half a day with two spacecrafts) at the equator also characterizes this instrument [34].

Table 1 details the SLSTR bands, highlighting in gray those at 500 m spatial resolution used to detect volcanic thermal features in the SWIR spectral region (e.g., [27]).

### 3.2. The Normalized Hotspot Index—NHI_SWIR_

The normalized index in Equation (1) enables the identification and mapping of high-temperature targets [29] by analyzing the TOA (top of the atmosphere) radiances measured in the SWIR band, at 1.6 µm ($L_{SWIR1}$) and 2.2 µm ($L_{SWIR2}$):

(1) $${NHI}_{SWIR}=\frac{L_{SWIR2}-L_{SWIR1}}{L_{SWIR2}+L_{SWIR1}}$$

**Figure 3:**
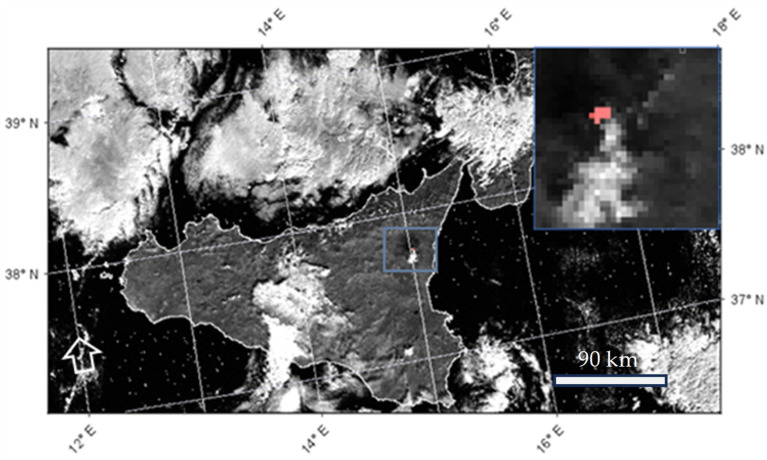
Lava flows (red pixels) from Mt. Etna (Sicily, Italy) eruption of 1 January 2023 detected analyzing values of NHI_SWIR_ > 0. The zoom of the Mt. Etna area is shown on the top right-hand corner.

In previous studies, we showed that positive values of the NHI_SWIR_ usually characterize hot targets such as lava flows and lava lakes [29,33]. The NHI algorithm exploits this index behavior to map the above-mentioned features on a global scale, through L8/9 OLI/OLI2 and S2-MSI data (up to 20 m spatial resolution), under the GEE (Google Earth Engine) platform, and provides automated notifications about volcanoes with evidence of thermal activity recorded over the previous 48 h [36]. Another normalized index, analyzing the TOA radiances in the NIR (near-infrared) and SWIR1 bands, is then used to improve the mapping of more intense thermal features [29]. The NHI_SWIR_ index was also used with success to infer information about changes in thermal activity at the Ambrym (Vanuatu), Kilauea, and Mauna Loa (Hawaii, USA) volcanoes, using Himawari-8 AHI (Advanced Himawari Imager) and GOES-R ABI (Advanced Baseline Imager) data at low-spatial/high-temporal resolution (e.g., [37,38]). These studies showed that negative values of the NHI_SWIR_ index should be explored to retrieve more accurate information about volcanic thermal anomalies by means of coarse spatial resolution data. Hence, the analysis of negative values of the NHI_SWIR_ index may allow for a more effective identification of these features even when SLSTR SWIR data are used. Figure 3 displays the thermal anomaly map generated from the daytime SLSTR data of 1 January 2023 covering the Mt. Etna (Sicily, Italy) area. By considering “hot” the pixels with values of NHI_SWIR_ > 0, lava flows from Mt. Etna were identified without generating false detections also thanks to the use of a spectral filter on S6 radiance [29] and despite the presence of significant cloud cover.

**Figure 4:**
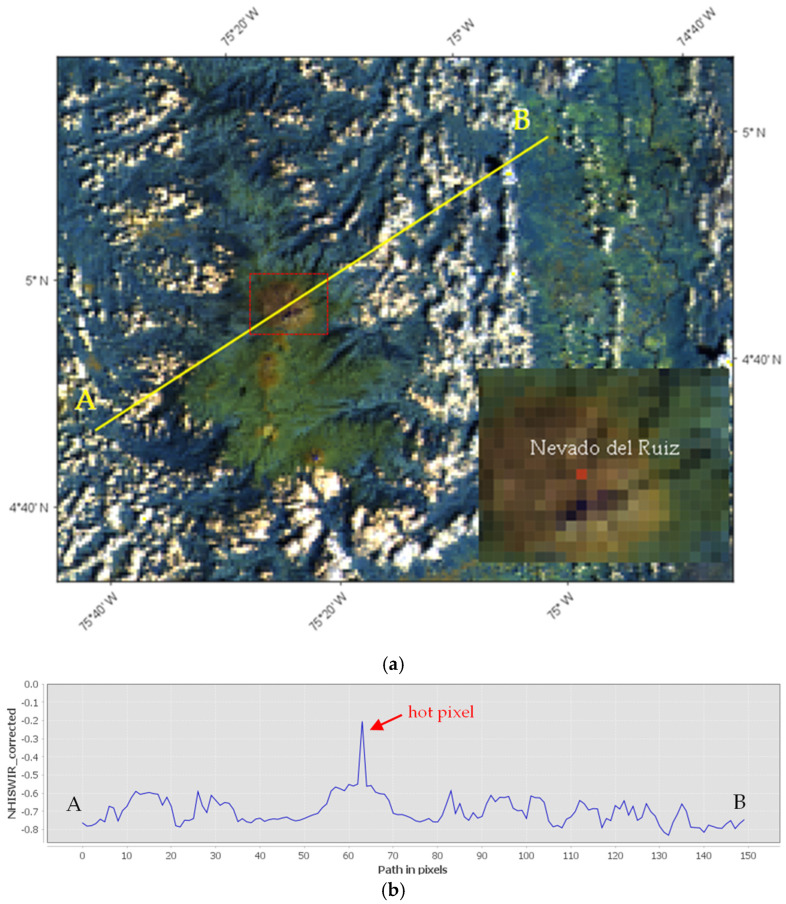
(**a**) SLSTR RGB (Red = B6; Blue = B5; Green = B3) subset image of Nevado del Ruiz (Colombia) volcano (see area marked by the dotted red line magnified in the top panel) of 5 January 2024 at 15:04 UTC showing a thermal anomaly, in red, intersected by the A-B transect (yellow line) and associated with a moderate thermal volcanic activity; (**b**) spatial profile of the NHI_SWIR_ retrieved along the same transect, showing the increment of the index up to −0.20 just in correspondence to the hot pixels located over the Nevado del Ruiz crater.

A moderate thermal volcanic activity, characterizing for instance the Nevado del Ruiz (Colombia) on 5 January 2024 [39], required, however, the analysis of negative values of the index to be identified. Figure 4 displays the RGB (Red = S6; Green = S5; Blue = S3) map from the Sentinel 3-SLSTR scene of 15:04 UTC (Figure 4a) and the spatial profile of the NHI_SWIR_ index retrieved along the A–B transect (Figure 4b). The thermal anomaly affecting the crater area (see red pixel) increased the index value above that of the background, up to −0.2, remaining negative.

Starting with this evidence, we assessed the contribution of daytime SLSTR SWIR data in characterizing four eruption phases of the Home Reef volcano, which is located in a remote area of the Pacific Ocean (see Section 2), where satellite observations represent a very important source of information [40]).

### 3.3. NHI Implementation on SLSTR SWIR Data

To investigate the Home Reef activity, we analyzed all the diurnal Sentinel 3-SLSTR L1B data, covering the area between 18÷22 S in latitude and 172÷177 W in longitude, available during the first phase (1 September 2022–5 October 2022), the second phase (1 September 2023–30 November 2023), the third phase (1–31 January 2024), and the fourth phase (1 June 2024–31 August 2024) of the Home Reef eruption.

In more detail, we downloaded those data from the EUMETSAT Data Store [41], by extracting the S5 and S6 TOA radiances, under nadir view, on a 0.5 km grid, together with the corresponding geographical coordinates and flags concerning the cloud presence based on specific threshold tests [42]. Two detector stripes (A and B) are defined for SLSTR [43] and always delivered with the L1B data, unlike the C stripe, which is a combination of both [44]. Indeed, as described in detail in [45], SWIR channels detectors have another extra column of four active elements along the scan direction to enable the acquisition of the same target with two different pixels. In this work, we used only the A stripe (bands S1-S6), with the B stripe, which refers only to SWIR channels (band S4 to S6) [43], which was analyzed mainly to verify possible differences with results retrieved using the A stripe (see the Discussion Section).

Hence, we performed a pre-processing phase to correct the S5 and S6 TOA radiances through the suggested adjustment factors based on vicarious calibration analysis (e.g., [46,47,48]). Afterward, the NHI_SWIR_ index was computed on each analyzed daytime scene by retrieving its maximum value within a 5 × 5-pixel box centered around the Home Reef location (18.990627 S, 174.763718 W; see Figure 1) also verifying the presence of clouds over the target area. The information from cloudy data was retrieved from the cloud masking product detailed in [44]. Moreover, we performed a visual inspection of the satellite imagery and analyzed the percentage of cloud cover through the Sentinel Hub EO Brower (https://apps.sentinel-hub.com/eo-browser/, accessed on 15 January 2025).

## 4. Results

### 4.1. Investigating the First Home Reef Eruption Phase

**Figure 5:**
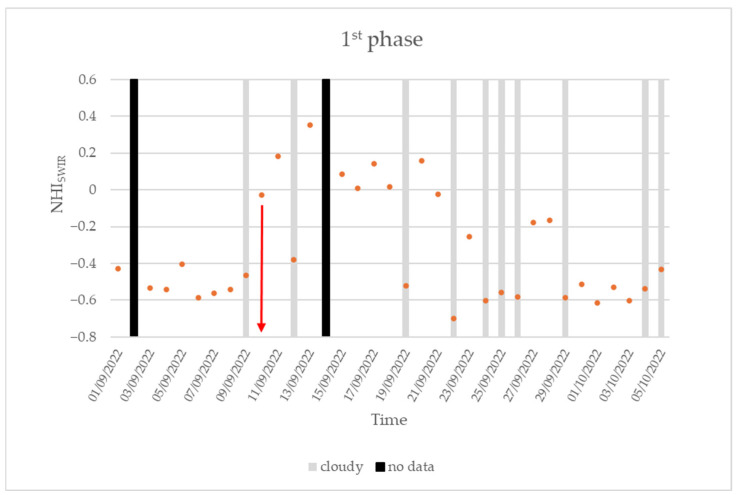
Time series of the maximum NHI_SWIR_ value (orange dots) retrieved over the Home Reef’s area from daytime Sentinel-3 SLSTR data of 1 September–5 October 2022 with indication of cloudy data (marked in gray) and no data (marked in black). Red arrow, on 10 September, marks the first significant increment in the maximum index value.

Figure 5 displays the time series of the maximum NHI_SWIR_ index value (see orange dots) retrieved over the Home Reef area during the period 1 September–6 October 2022, referring to the first phase of the subaerial eruption. Cloudy days and no data are marked in gray and black, respectively.

The figure shows that, in between 1 and 9 September 2022, the NHI_SWIR_ index was always negative, regardless of cloud coverage, with values mostly lower than −0.4. On 10 September, a first significant increment in the maximum index value was recorded (see red arrow). Specifically, the index increased up to −0.03 from the SLSTR scene of 20:48 UTC (in cloud-free conditions), in agreement with the start of a lava effusion automatically detected by the NHI system from the Sentinel-2 MSI overpass of the day before at 21:59 UTC, with the thermal anomaly, which covered an area of about 8800 m^2^ (note that the daytime SLSTR data of 9 September at 21:14 UTC was cloudy over the target area).

**Figure 6:**
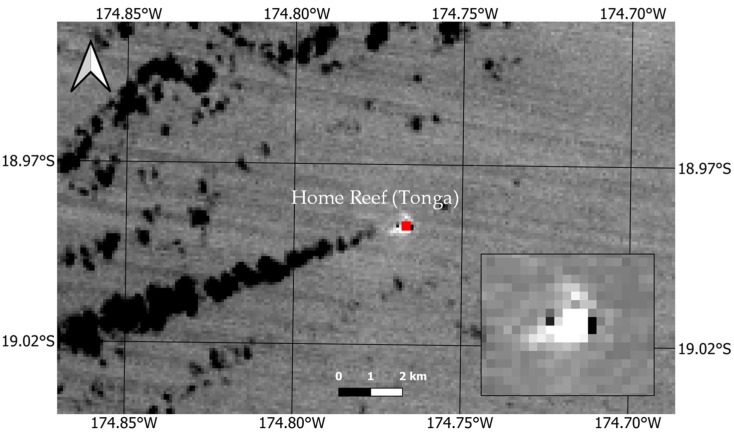
ASTER TIR (band 13; 10.25–10.95 µm) image (90 m spatial resolution) of 14 September 2022 at 21:45 UTC showing in red the thermal anomaly detected by RASTer [50] over the Home Reef area. In the inset, zoom of the target area, with the bright pixels associated with higher values of the brightness temperature measured in band 13 (10.25–10.95 µm) because of lava effusion.

In the following days, the index further increased, reaching positive values, suggesting the occurrence of a more intense lava effusion, which continued to expand the island [49]. The Terra-ASTER data of 14 September, analyzed through the RASTer system detailed in [50], show that an extended thermal anomaly (of about 24300 m^2^ based on information from the NHI tool) affected the Home Reef area the day after the peak of thermal volcanic activity marked by the maximum index value (NHI_SWIR_ = 0.35) in Figure 6. This is indicated by the “anomalous” pixels (in red) detected within the region magnified on the bottom-right side of Figure 6 and mostly corresponding to the bright ones, which were associated with higher brightness temperature values (because of lava effusion) measured in ASTER band 13 displayed in the background.

During the period 15–21 September 2022, the NHI_SWIR_ was still positive except when meteorological clouds strongly affected the target area. Afterward, the maximum index value progressively decreased, suggesting an intensity reduction in the lava effusion, despite a slight increment (up to values of −0.2) occurring in between 27 and 28 September 2022. In the following days, index values lower than −0.4 were recorded (see early October 2022). They were comparable to those recorded before the lava effusion also in cloud-free conditions (see 1–8 September 2022), suggesting the end of first eruption phase.

### 4.2. Investigating the Second Phase of the Home Reef Eruption

**Figure 7:**
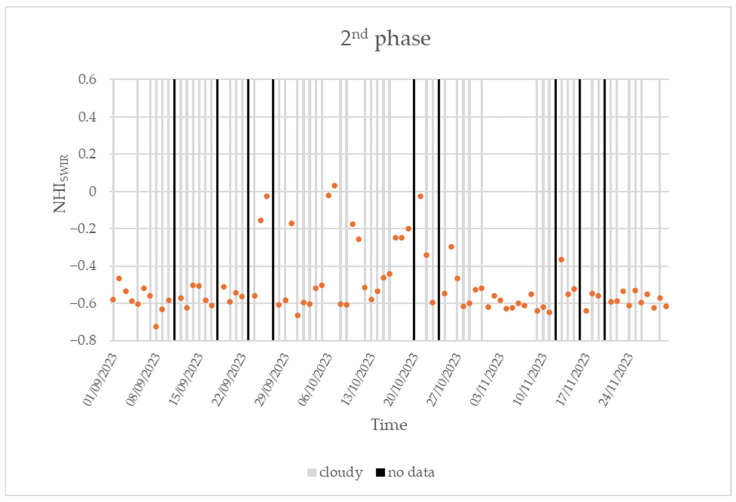
Time series of the maximum NHI_SWIR_ index value (orange dots) retrieved over the Home Reef’s area from daytime Sentinel-3 SLSTR data of 1 September 2023–30 November 2023. Cloudy data and no data are marked in gray and black, respectively.

After the eruptive events analyzed in the previous section, and a sporadic eruption flagged by the NHI system on 12 May 2022 [49], the second phase of the Home Reef eruption occurred. Figure 7 shows the time series of the maximum NHI_SWIR_ index value retrieved from the daytime SLSTR SWIR data of 1 September 2023–30 November 2023.

The figure shows that, until 24 September 2022, values of −0.7 < NHI_SWIR_ < −0.5 were mostly recorded. The lowest value of the analyzed indicator characterized the daytime SLSTR scene, showing a percentage of clouds estimated around 80%. On 25 September, the NHI_SWIR_ index increased up to −0.16, marking the start of the second eruption phase.

Based on information inferred from Figure 7, the peak in the lava effusion was reached on 7 October 2023, when the NHI_SWIR_ increased to slight positive values. The comparison with the plot in Figure 4 indicates, however, the occurrence of a less intense thermal volcanic activity than in the first eruption phase, in agreement with the results of a previous study [49]. In the following days, the maximum NHI_SWIR_ index fluctuated around values of −0.2 (e.g., see 10–19 October), and, after a new increment occurring on 21 October, values lower than −0.4 were recorded also in cloud-free conditions. These values, characterizing the SLSTR scenes acquired after 26 October, marked the end of the second eruption phase.

### 4.3. Investigating the Third and Fourth Phases of the Home Reef Eruption

**Figure 8:**
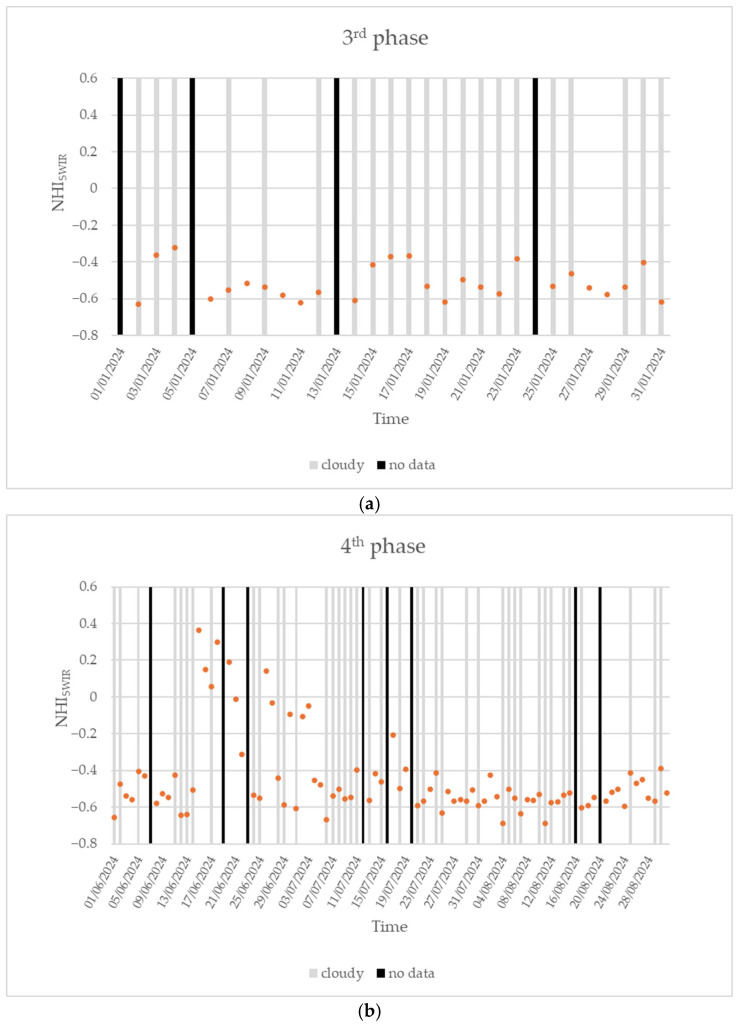
Time series of the maximum NHI_SWIR_ index value (orange dots) retrieved over the Home Reef’s area from daytime Sentinel-3 SLSTR data of (**a**) January 2024; (**b**) 1 June 2024–31 August 2024. Cloudy data and no data are marked in gray and black, respectively.

In January 2024, the third Home Reef eruption phase occurred [49]. The presence of an almost continuous cloud coverage over the target area did not enable, however, the analysis of this eruptive phase through daytime SLSTR data (see Figure 8a). On the other hand, the following eruption was better investigated as shown in Figure 8b, displaying the time series of the NHI_SWIR_ index retrieved over the period 1 June 2024–31 August 2024.

The plot shows that, until mid-June 2024, the NHI_SWIR_ index was always negative, regardless of cloud coverage, indicating the absence of ongoing thermal activity. Starting from 15 June 2024, the index abruptly increased to positive values, marking the occurrence of the fourth eruption phase [49]. In the following days, the analyzed indicator fluctuated around positive values due to the lava effusion, which appeared progressively less intense since the third week of June. After the 24–25 June 2024 cloudy period, a new increment in the maximum NHI_SWIR_ index value was recorded. The analyzed indicator assumed slight negative values in the following days, suggesting the occurrence of a moderated effusive activity (see period 27 June–3 July 2024). After the strong decrease (below values of −0.4) recorded since 4 July, the maximum index value once again increased up to −0.2 on 17 July 2024. This new increment was consistent with the thermal anomaly detected three days after by the NHI tool using S2-MSI data. The fourth eruption phase ended in August, when it was, however, much less intense and continuous (e.g., as indicated by MIROVA from the VIIRS data). It is worth noting that, during the fourth eruption phase, the NHI_SWIR_ index increased up to about 0.36 as in the first eruption phase (see Section 4.1). This similarity seems to indicate that the first and fourth eruption phases were comparable in terms of intensity level, despite the different time durations [49].

## 5. Discussion

The results shown in this work are consistent with the outcomes of a recent study performed by using a multi-sensor approach investigating the space–time evolution of the island that formed during the Home Reef eruption [49].

To better assess information inferred from the time series of the NHI_SWIR_ index, we analyzed the FIRMS products generated from the daytime VIIRS/Suomi-NPP and VIIRS/NOAA-20 data. In detail, we calculated the total FRP (FRP_TOT_) for each daytime scene, by summing the FRP of the hot pixels detected over the target area. In the presence of more than one daytime overpass, the maximum value of FRP_TOT_ was considered.

**Figure 9:**
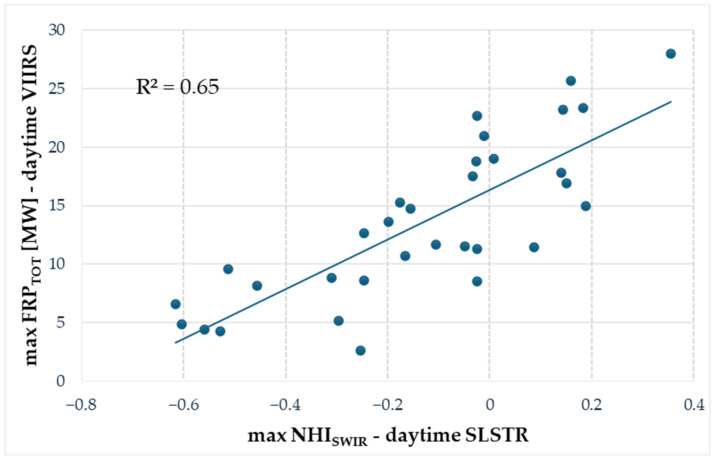
Scatter plot between maximum NHI_SWIR_ and the maximum total FRP (FIRMS) retrieved from daytime SLSTR and VIIRS data, respectively, during the different Home Reef eruption phases analyzed in this work. The plot does not include the maximum NHI_SWIR_ values associated with cloudy data.

Figure 9 displays the scatter plot between the maximum NHI_SWIR_ value (from daytime SLSTR data) and the maximum value of total FRP estimated using FIRMS information and retrieved from the daytime VIIRS data. The plot shows a good agreement between the variables recorded during the different Home Reef eruption phases and the significance of the data sample, as indicated by an *R^2^* = 0.65 and a *p*-value = 2.43 × 10^−8^, respectively.

It is worth mentioning that, although the correlation analysis was probably affected by sub-pixel effects due to meteorological clouds and volcanic plumes, affecting the values of the NHI_SWIR_ index (e.g., on 19 September 2022, when FIRMS marked the peak of FRP_TOT_ around 61 MW, the daytime SLSTR data was cloudy, and the NHI_SWIR_ was equal to −0.52), the increasing/decreasing trends in the lava effusion inferred from the SLSTR SWIR observations were consistent with the information provided by FIRMS. As an example, the FRP_TOT_ increased from 18.82 MW on 10 September 2022 up to 41.25 MW on 14 September 2022, corroborating the increment of the NHI_SWIR_ index recorded during 10–13 September 2022 (see first eruption phase in Figure 5).

During the eruption’s second phase, the peak of FRP_TOT_ from the daytime VIIRS data (21 October 2023) was lower and equal to 22.7 MW. Since the first days of November 2023, the FRP_TOT_ decreased below 5 MW, and the index assumed values mostly lower than −0.5.

Regarding the third phase, only one daytime VIIRS observation provided information about the FRP_TOT_ (3.79 MW) on 22 January 2024, when, however, the SLSTR SWIR data were strongly affected by clouds (see Figure 8a). During the fourth eruption phase, the FRP_TOT_ corroborated information from Figure 8b, indicating that the lava effusion was comparable, in terms of intensity level, with that in the first eruption phase occurring in September–October 2022 (see [49]).

This analysis confirms that the NHI_SWIR_ index may be used to analyze the different phases of effusive eruptions, despite the sensitivity limits ascribable to the use of SWIR data. Since negative values of the NHI_SWIR_ index can then be explored to better investigate lava effusions, we adapted the NHI algorithm to the daytime SLSTR SWIR data by assessing its potential in detecting volcanic thermal features from a moderate-to-high intensity level.

The algorithm configuration analyzing values of NHI_SWIR_ > −0.2 and using a spectral test on S6 radiance to filter out possible spurious effects appeared as the most suited for this purpose. Indeed, although lower critical values of the index (e.g., NHI_SWIR_ > −0.3) seem to be more sensitive to moderate thermal activities (see Section 4), the above-mentioned algorithm configuration minimized the occurrence of false detections over sea areas when the SLSTR data were analyzed (i.e., 294 satellite imagery of 3000×2400 pixels in size).

**Figure 10:**
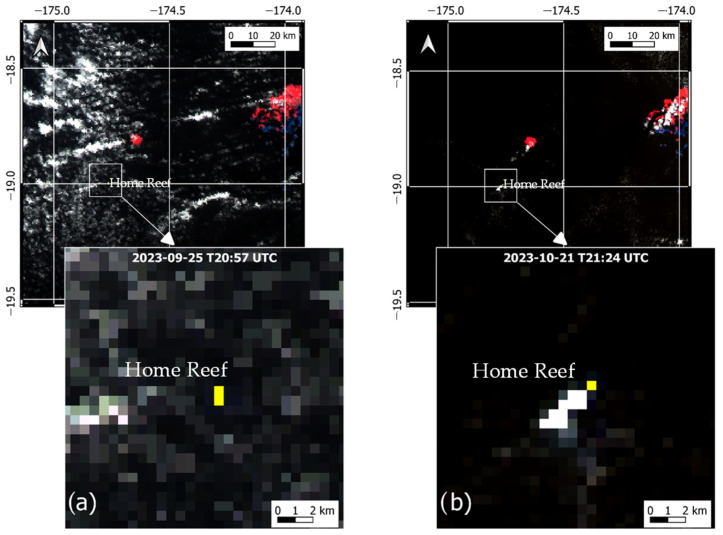
Thermal anomaly maps from daytime Sentinel-3 SLSTR data showing, in yellow, hot pixels (i.e., NHI_SWIR_ > −0.2 *AND* L_SWIR2_ > 0.5 mW/m^2^ sr nm) associated with the Home Reef eruption; (**a**) 25 September 2023, at 20:57 UTC; (**b**) 21 October 2023, at 21:24 UTC. In the background, the RGB (Red = S6; Green = S5; Blue = S3) image.

In Figure 10, we show an example of thermal anomaly maps generated using the NHI algorithm configuration described above. In more detail, the figure displays the thermal anomaly (in yellow) detected on daytime SLSTR scenes of 25 September 2023 at 20:57 UTC (Figure 10a) and 21 October 2023 at 21:24 UTC (Figure 10b), which were acquired during the second and less intense phase of the Home Reef eruption. The algorithm detected the thermal anomaly over the Home Reef area without generating false detections despite the presence of significant and extended cloud coverage. Moreover, by using the same algorithm configuration, a thermal anomaly was correctly also identified over the Tofua volcano (Tonga Islands) on 15 September 2022 and 6 September 2023, as indicated by the manual inspection of the SLSTR imagery. This feature was also consistent with the information from other systems (e.g., FIRMS on 6 September 2023).

We then compared the outputs of the used NHI algorithm configuration with the SLSTR L2 FRP product [48]. The latter was downloaded (from EUMETSAT) by selecting the area located in between 18 ÷ 22 S in latitude and 172 ÷ 177 W in longitude [51], for both the first (9 September 2022–5 October 2022) and second phase (23 September 2023–30 November 2023) of the eruption.

**Figure 11:**
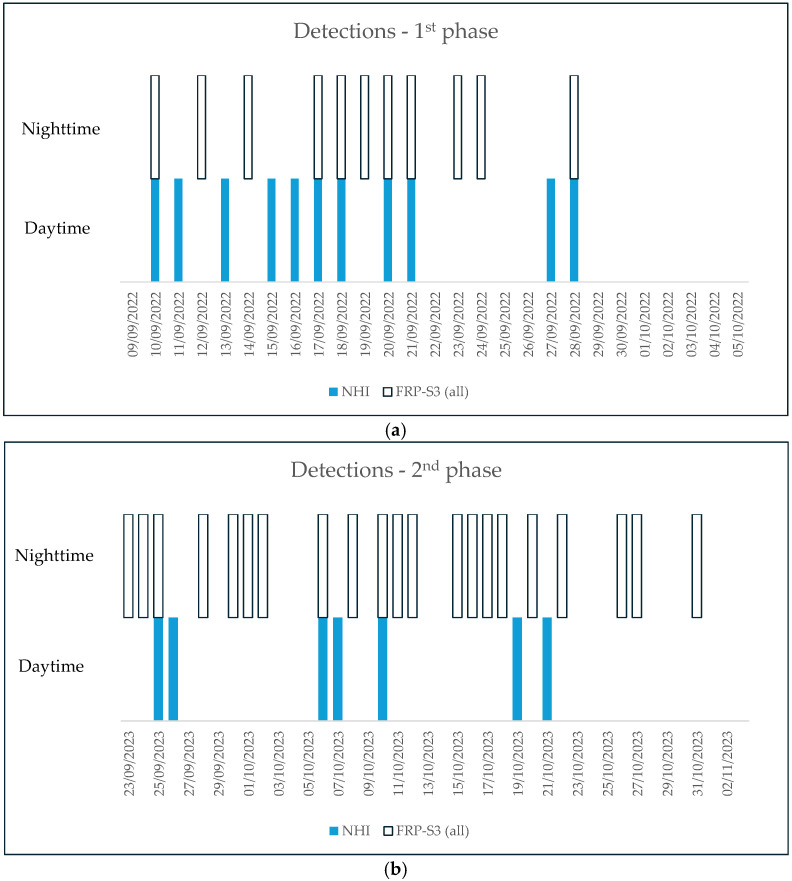
Integration of daytime (NHI) and night-time SLSTR (SLSTR FRP product) over the Home Reef area: (**a**) first eruption phase; (**b**) second eruption phase.

The SLSTR FRP product from the SWIR data is only generated at night-time [52] and is available by day only for ocean gas flares [53]. Therefore, we did not find any information from the SLSTR FRP product over the Home Reef area in daylight conditions, while estimates of the FRP from the night-time SLSTR SWIR data were available. Figure 11 shows that, by integrating the hotspot detections from the NHI and SLSTR L2 FRP product (see empty and blue bars), the Home Reef activity could be monitored in a more efficient way (i.e., in both daylight and night-time conditions). This integration was particularly significant, especially during the first eruption phase, when the NHI algorithm configuration provided unique information about the Home Reef activity on several days of the eruption (e.g., see Figure 11a). During the second eruption phase, meteorological clouds affected, in a more significant way, the identification of the thermal anomaly over the Home Reef area in daylight conditions.

**Figure 12:**
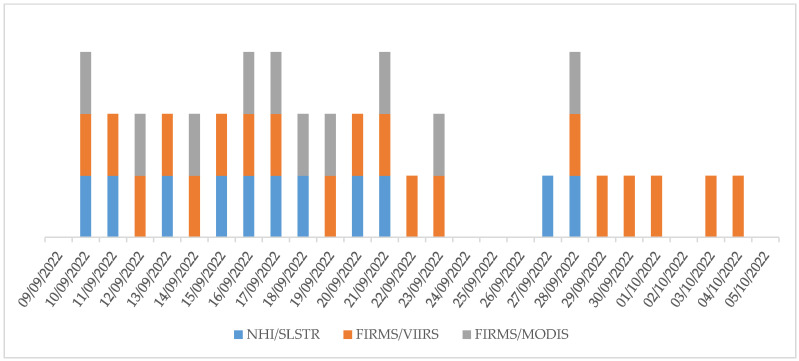
Daytime SLSTR (blue bars; NHI), VIIRS (orange bars; FIRMS), and MODIS (gray bars; FIRMS) data with evidence of thermal anomalies over the Home Reef area during the first phase of the Home Reef eruption.

The contribution of NHI detections to the monitoring of Home Reef activity is also evident for comparison with FIRMS. Figure 12 shows that combining the SLSTR, VIIRS, and MODIS observations, the number of daytime hotspot detections during the first phase of the eruption increased (see blue, gray, and orange bars). It is interesting to note that the first evidence of a thermal anomaly over the Home Reef area was provided by the three sensors on 10 September 2022. Moreover, neither NHI nor FIRMS provided information about the Home Reef activity during 24–26 September 2022, because of cloudy conditions. In addition, while FIRMS-VIIRS provided unique information about the less intense thermal anomaly characterizing the Home Reef activity at the end of first eruption period (see 30 September 2022–5 October 2022), the NHI-SLSTR algorithm was the only one providing information about the Home Reef activity on 27 September 2022 (see blue bars).

The number of days with NHI-SLSTR detections was comparable to that for FIRMS-MODIS and lower than that for FIRMS-VIIRS. On the other hand, VIIRS guarantees up to four observations per day, with an increased probability of detecting volcanic thermal features in clear-sky conditions. Moreover, thanks to the MIR bands at 375 m spatial resolution, this sensor enables better identification of subtle hotspots (e.g., [17]).

**Figure 13:**
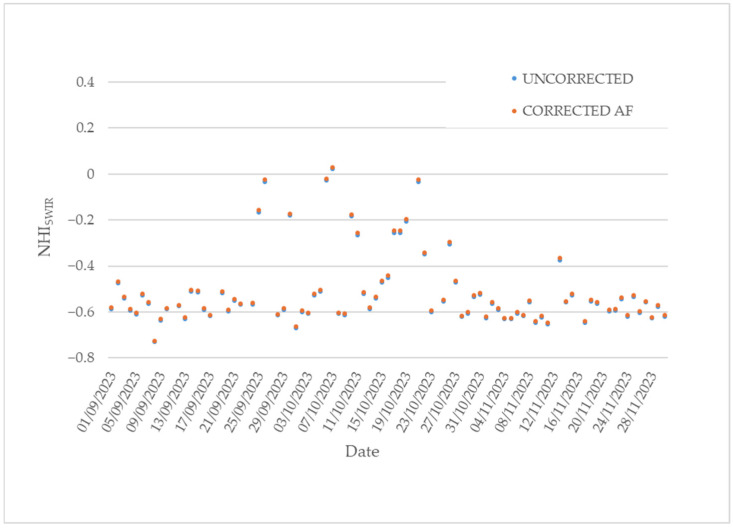
Maximum NHI_SWIR_ index value retrieved during the second phase of the Home Reef eruption, without (blue dots) and with (orange dots) correction for SWIR radiance.

Regarding the factors affecting the SLSTR SWIR observations, we observed a slight and systematic increment of the NHI_SWIR_ index value when the corrected radiances were analyzed (see Figure 13). It is worth mentioning that, although we used only the A stripe (see Section 3.3), the B stripe could integrate the results of the thermal anomaly identification. We observed, for instance, the identification of two additional hot pixels over the Home Reef area from the B stripe on 13 September 2022. These additional hot pixels better detailed the thermal anomaly in terms of spatial extent. Nonetheless, this aspect needs to be deeply investigated before integrating information from the two stripes operationally.

Meteorological clouds had, however, the greatest impact on the results of this work. These features, on the one hand, did not generate false detections and, on the other hand, partially or completely obscured the underlying hot targets. It should be stressed that the cloud mask embedded within the SLSTR L1 product [42,54] did not always provide reliable information about the cloudy pixels, as confirmed by the manual inspection of the satellite imagery. In this circumstance, we interpreted the SLSTR data as cloud free also considering that the NHI_SWIR_ values were coherent with those retrieved in the presence of thermal anomalies over the target area. This aspect should be taken into consideration when SLSTR data are analyzed to infer information about changes in the lava effusion, as performed in this work.

## 6. Conclusions

In this study, we assessed the potential of daytime SLSTR SWIR observations in providing information about high-temperature volcanic features through time series of the maximum NHI_SWIR_ index value, computed over the Home Reef area during four different eruption phases. The results show that the used indicator is sensitive to the increasing/decreasing trends in the lava effusion.

The NHI algorithm configuration adapted to daytime SLSTR SWIR data integrated information from the operational SLSTR L2 FRP product and complemented the FIRMS detections in daylight conditions, retrieved from MODIS and VIIRS data at higher temporal resolution, despite some limitations (e.g., lower sensitivity to less intense thermal activities).

To improve the identification of high-temperature volcanic features over land areas, by exploiting lower critical values of the NHI_SWIR_ index, which are more sensitive to moderate eruptions, the use of a land–sea mask is suggested. Moreover, the NHI_SWNIR_ could be used jointly with the above-mentioned index to better characterize thermal anomalies of a very high intensity level (e.g., [29]).

The analysis of night-time Sentinel 3-SLSTR observations is also under evaluation, with the aim of developing an automated processing chain capable of supporting the monitoring of active volcanoes in NRT through the analysis of SWIR observations and also through the estimates of FRP starting from NHI detections. This system could contribute to the surveillance of remote volcanic areas, where multi-sensor satellite systems may better address the lack of traditional ground-based systems.

## Figures and Tables

**Table 1:** SLSTR spectral channels: in gray, the SWIR channels.

Band	Central Wavelength (nm)	Spatial Resolution (m)
S1	554.27	500
S2	659.47	500
S3	868	500
S4	1374.80	500
S5	1613.40	500
S6	2255.60	500
S7	3742	1000
S8	10854	1000
S9	12,022.50	1000
F1	3742	1000
F2	10,854	1000

## Data Availability

Sentinel-3 SLSTR data analyzed in this work were downloaded from the Copernicus Browser https://browser.dataspace.copernicus.eu/ (accessed on 15 January 2025).
